# Predictors of Complete Remission in Differentiated Thyroid Cancer: A Study from the Nuclear Medicine Department of Ibn Rochd Hospital in Casablanca, Morocco

**DOI:** 10.4314/ejhs.v35i5.5

**Published:** 2025-09

**Authors:** Hajar Tabiti, Karima Bendahhou, Amal Guensi

**Affiliations:** 1 Laboratory of Cellular and Molecular Inflammatory, Degenerative and Oncologic Pathophysiology, Faculty of Medicine and Pharmacy, Hassan II University, Casablanca, Morocco; 2 Department of Epidemiology and Public Health, Ibn Rochd Hospital Casablanca Cancer Registry, Casablanca, Morocco; 3 Nuclear Medicine Department, Ibn Rochd Hospital Casablanca, Morocco

**Keywords:** Complete remission, Thyroid cancer, Differentiated thyroid carcinoma, Multivariate analysis, Prognostic factors

## Abstract

**Background:**

Differentiated thyroid cancers (DTCs) are the most common endocrine malignancies. Although they are generally associated with a favorable prognosis, some patients do not achieve complete remission after treatment. This study aimed to identify predictive factors for complete remission among Moroccan patients with DTCs.

**Patients and Methods:**

We retrospectively reviewed the medical records of patients diagnosed with DTCs between 2004 and 2012 and followed at the Nuclear Medicine Department of Ibn Rochd Hospital. Associations between complete remission and various epidemiological and histopathological factors were assessed using the chi-square test and logistic regression.

**Results:**

A total of 1,366 patients were included. The cohort comprised 89.6% females and 10.4% males, with a sex ratio of 8.5. The mean age at diagnosis was 44 years (range: 14–85 years), and the mean follow-up duration was 125 months. Logistic regression analysis identified the following as significant predictors of complete remission: female sex, age <55 years, papillary thyroid carcinoma, tumor size ≤4 cm, unilaterality, absence of extrathyroidal extension, absence of vascular invasion, undetectable postoperative thyroglobulin (TG) levels, and absence of local or distant involvement.

**Conclusion:**

Younger age, female sex, favorable histology, and limited tumor burden were associated with a higher likelihood of complete remission in Moroccan patients with DTCs. Identifying these factors is crucial for tailoring treatment strategies and optimizing follow-up, particularly for patients at higher risk of persistent or recurrent disease.

## Introduction

Differentiated thyroid carcinomas (DTCs), which account for approximately 19% of all thyroid cancers, are classified into papillary thyroid carcinomas (PTCs), follicular thyroid carcinomas (FTCs), and oncocytic cell carcinomas (OCCs). These cancers generally carry an excellent prognosis, with 10-year survival rates of 97% for PTCs and 75% for FTCs (1,2).

Over the past three decades, the global incidence of thyroid cancer (TC) has risen markedly (3). In the United States, for example, the incidence nearly tripled, increasing from 4.9 to 14.3 per 100,000 individuals, with most cases being small, non-palpable DTCs associated with excellent outcomes. The rise has been more pronounced in women than in men (4,5). Similar trends were observed in the Casablanca Cancer Registry, where the incidence increased from 6.6/100,000 in 2008–2012 to 8.0/100,000 in 2013-2017, again with a strong female predominance (6).

Surgery remains the cornerstone of treatment for DTCs, usually complemented by radioactive iodine (RAI) therapy to eradicate residual thyroid tissue and treat locoregional or distant metastases. Post-treatment follow-up typically involves physical examination, cervical ultrasound, and laboratory testing—including thyroglobulin (TG), anti-thyroglobulin antibodies, and thyroid-stimulating hormone (TSH) levels—along with whole-body iodine scintigraphy. Complete remission (CR) is defined as the absence of structural or biochemical evidence of disease (7,8).

International studies have identified age, sex, histological subtype, histoprognostic features, lymph node involvement, and metastases as key determinants of complete remission (8,9). However, research in Morocco remains limited. Given potential differences in tumor biology and genetic predisposition among North African populations, evaluating these predictive factors in Moroccan patients is essential.

This study therefore aimed to identify predictors of complete remission in Moroccan patients with DTCs followed at the Nuclear Medicine Department of Ibn Rochd hospital in Casablanca between 2004 and 2012.

## Patients and Methods

This analytical cross-sectional study retrospectively evaluated factors predictive of complete remission in DTCs. Eligible patients underwent total thyroidectomy and were followed in the Nuclear Medicine Department between 2004 and 2012. Patients with incomplete records or with non-DTC thyroid cancers were excluded.

**Follow-up procedures**: All patients underwent total thyroidectomy. Postoperative TG levels were measured three months after surgery, following levothyroxine withdrawal to assess residual disease and determine the need for RAI therapy. The American Thyroid Association (ATA) guidelines were followed for RAI ablation. Doses ranged from 1.11 to 3.7 GBq, with 3.7 GBq administered in cases of suspected residual tumor. For metastatic disease, the dose varied between 3.7 and 7.4 GBq. In children and adolescents, we used European Association of Nuclear Medicine (EANM) recommendations.

TG was reassessed six months after RAI therapy and annually thereafter to monitor residual disease or recurrence. Treatment was repeated if necessary, with an interval of 3–6 months between iodine-131 administrations. Patients were classified as follows:

**Complete remission**: undetectable TG and no structural evidence of disease on imaging.

**Biochemical disease**: detectable TG (>1–≤10 ng/mL) without structural lesions.

**Persistent disease**: elevated TG with morphological evidence of disease.

**Statistical analysis**: Associations between CR and epidemiological, clinical, and histopathological factors were examined using chi-square and Fisher's exact tests. Logistic regression (stepwise method) identified independent predictors of CR. Statistical significance was set at p < 0.05. Analyses were conducted using JAMOVI 2.3.17.

**Ethical considerations**: The study was approved by the Biomedical Research Ethics Committee and the Ethics Committee of Ibn Rochd University Hospital, Casablanca (Approval Nos. 02/2022 and 14/22).

## Results

A total of 1,366 patients were included in this study, with a mean follow-up duration of 125 months (range: 10 months to 15 years). Women comprised the majority of the cohort (89.6%), resulting in a male-to-female ratio of 1:8.5. Patients younger than 55 years accounted for 79% of cases, while those aged 55 years and older represented 21%. All patients underwent total thyroidectomy. The mean TG level was 131 ng/ml (range: 0.01–81,007 ng/ml; SD: 2360 ng). PTC was the predominant histological type, representing 93% of cases, while FTC accounted for 7%. Tumors were unilateral in 82% of patients and bilateral in 18%. Tumor encapsulation was observed in 21% of cases. Tumors measuring ≤4 cm were present in 86.4% of patients, whereas 13.6% had tumors >4 cm. Multifocality, nodular capsule invasion, extrathyroidal extension, and vascular invasion were reported in 28.7%, 14%, 5.38%, and 6% of cases, respectively (Table 1).

**Table 1:** Characteristics of patients

Variable	Percent
**Age**	
<55 years	78.7
≥55 years	21.2
**Gender**	
Male	10.4
Female	89.6
**Histological type**	
Papillary thyroid carcinoma	93.0
Follicular thyroid carcinoma	7.0
**Tumor size**	
≤ 4 cm	86.4
>4 cm	13.6
**Encapsulated**	
Yes	21.6
No	78.4
**Bilateral**	
Yes	18.1
No	81.9
**Multifocality**	
Yes	28.7
No	71.3
**Nodular invasion**	
Yes	14.2
No	85.8
**Extrathyroidal extension**	
Yes	5.3
No	94.6
**Vascular invasion**	
Yes	6.1
No	93.8
**TG postoperative levels**	
Undetectable	13.0
Detectable (> 1 ng/ml et≤10 ng/ml),	56.0
>10 ng/ml	31.0
**Lymph node involvement at diagnosis**	
Yes	2.6
No	97.4
**Distant metastasis at diagnosis**	
Yes	0.6
No	99.4
**Stage**	
I	94.1
II	4.59
III	0.68
IV	0.60
**Risk group (ATA 2015)**	
Low Risk	83.0
Intermediary risk	9.0
High risk	8.0
**Radioiodine therapy**	
Yes	87.0
No	13.0
**TG level after Radioiodine**	
Undetectable	82.0
Detectable (>1 ng/m and≤10 ng/ml)	13.0
>10 ng/ml	5.0

Patients were stratified into three groups based on postoperative TG levels and their response to radioactive iodine therapy. With regard to postoperative TG, 13% had undetectable levels (<1 ng/ml), 56% had detectable levels (>1–≤10 ng/ml), and 31% had TG >10 ng/ml. At diagnosis, 2.58% of patients presented with cervical lymph node involvement, and 0.65% had distant metastases, predominantly in the bones (87.5%) and lungs (12.5%). According to the 8th edition of the AJCC staging system, most patients (94.13%) were classified as Stage I, followed by 4.59% in Stage II, 0.68% in Stage III, and 0.6% in Stage IV. Based on the ATA risk stratification, 83% were categorized as low risk, 9% as intermediate risk, and 8% as high risk (Table 1).

Regarding RAI therapy, 87% of patients received an ablative dose, with 94.64% requiring a single course, 3.82% requiring two courses, and 1.29% requiring three or more. The mean iodine activity administered was 3.996 GBq (range: 3.7–27.75 GBq). The mean delay before initiating RAI therapy was 18 months (range: 30 days–37 months). Following RAI therapy, TG levels were undetectable in 82% of patients, detectable (>1–≤10 ng/ml) in 13%, and >10 ng/ml in 5%. At the end of follow-up, 85% of patients were in complete remission, 7% had biochemical disease, and 8% had persistent disease.

Univariate analysis identified significant associations between CR and several variables. Female patients had a significantly higher CR rate (89.9%) compared with males (66.9%; p < 0.001). Younger patients (<55 years) also had a higher CR rate (91%) compared with those aged ≥55 years (73.7%; p < 0.001). PTC was associated with a higher CR rate (88.2%) than FTC (77.3%; p = 0.023). Tumor characteristics also influenced CR; patients with tumors ≤4 cm achieved CR in 90.8% of cases, whereas those with tumors >4 cm had a CR rate of 70.9% (p < 0.001). Unilateral tumors were associated with a higher CR rate (88.1%) than bilateral tumors (82.4%; p = 0.007). Multifocal tumors were associated with lower CR (84.4%) compared with unifocal tumors (89%; p = 0.023), and encapsulated tumors achieved higher CR (91.4%) than non-encapsulated tumors (86.7%; p = 0.032) (Table 2).

**Table 2:** The univariate analysis for all factors included in the study

Factors	Complete remission %		p value

	No	Yes	
**Gender**			<.001
Female	10.1	89.9	
Male	33.1	66.9	
**Age**			<.001
<55 years	9.0	91.0	
≥55 years	26.3	73.7	
**Histological type**			0.023
PTC	11.8	88.2	
FTC	22.7	77.3	
**Tumor size**			<.001
≤4 cm	9.2	90.8	
>4 cm	29.1	70.9	
**Encapsulated**			0.037
Yes	8.6	91.4	
No	13.3	86.7	
**Bilateral**			0.007
Yes	17.6	82.4	
No	11.1	88.1	
**Multifocality**			0.023
Yes	15.6	84.4	
No	11.0	89	
**Nodular invasion**			<.001
Yes	25.3	74.7	
No	10.1	89.9	
**Extrathyroidal extension**			<.001
Yes	45.5	54.5	
No	10.4	89.6	
**Vascular invasion (embolus)**			<.001
Yes	33.3	66.7	
No	11.0	89.0	
**TG postoperative levels**			<.001
Undetectable	6.8	93.2	
Detectable	8.8	91.2	
>10 ng/ml	22.4	77.6	
**Cervical node involvement**			<.001
Yes	37.0	63.0	
No	11.8	88.2	
**Metastasis at Diagnosis**			<.001
Yes	80.0	20.0	
No	11.9	88.1	
**Stage**			<.001
I	10.4	89.6	
II	59.1	40.9	
III	85.7	14.3	
IV	75.0	25.0	

Factors associated with non-remission included nodular capsule invasion (CR: 74.7% vs. 89.9%; p < 0.001), vascular invasion (54.5% vs. 89.6%; p < 0.001), and extrathyroidal extension (66.7% vs. 89%; p < 0.001). Postoperative TG levels were also strongly associated with CR, with remission achieved in 93.2%, 91.2%, and 77.6% of patients with undetectable TG, detectable TG (>1–≤10 ng/ml), and TG >10 ng/ml, respectively (p < 0.001).

Lymph node involvement at diagnosis was linked to a lower CR rate (63%) compared with patients without lymph node involvement (88.2%; p < 0.001). Similarly, distant metastases were associated with a markedly reduced CR rate (20% vs. 88.1%; p < 0.001). Stage I patients achieved a significantly higher CR rate (89.6%) than those in Stages II (40.9%), III (14.3%), and IV (25%; p < 0.001) (Table 2).

Multivariate analysis identified several factors significantly associated with complete remission (p < 0.05). Female sex (OR: 3.57, 95% CI: 2.06-6.17, p < 0.001), age <55 years (OR: 1.84, 95% CI: 1.07–3.17, p = 0.026), papillary histology (OR: 1.34, 95% CI: 1.20–4.81, p = 0.040), tumor size ≤4 cm (OR: 2.35, 95% CI: 1.24–4.44, p = 0.008), and unilaterality (OR: 2.15, 95% CI: 1.33-3.46, p = 0.002) were predictive of remission. In addition, the absence of vascular invasion (OR: 2.25, 95% CI: 1.07–4.69, p = 0.031), absence of extrathyroidal extension (OR: 3.81, 95% CI: 1.58–9.16, p = 0.003), absence of lymph node invasion (OR: 3.35, 95% CI: 1.29-8.68, p = 0.024), and absence of distant metastases (OR: 18.56, 95% CI: 1.59–215.90, p = 0.020) were also strongly predictive of complete remission.

Postoperative thyroglobulin levels were significantly associated with remission. Patients with TG >10 ng/ml had lower odds of remission compared with those with undetectable TG (OR: 1.95, 95% CI: 1.09-3.49, p = 0.024). However, there was no significant association between disease stage and complete remission (p > 0.05) (Table 3).

**Table 3:** Multivariate analysis

Factor	Category	Odds Ratio	95% CI	p value
**Gender**	Male	1	–	–
	Female	3.57	[2.06–6.17]	<0.001
**Age**	≥55 years	1	–	–
	<55 years	1.84	[1.07-3.17]	0.026
**Histological type**	FTC	1	–	–
	PTC	1.34	[1.20-4.81]	0.040
**Tumor Size**	>4 cm	1	–	–
	≤4 cm	2.35	[1.24–4.44]	0.008
**Bilateral**	Yes	1	–	–
	No	2.15	[1.33–3.46]	0.002
**Vascular invasion**	Yes	1	–	–
	No	2.25	[1.07–4.69]	0.031
**Extrathyroidal extension**	Yes	1	–	–
	No	3.81	[1.58–9.16]	0.003
**TG postoperative**	Undetectable	1	–	–
	>10 ng/mL	1.95	[1.09–3.49]	0.024
**Cervical node involvement**	Yes	1	–	–
	No	3.35	[1.29–8.68]	0.013
**Metastasis at diagnosis**	Yes	1	–	–
	No	18.56	[1.59–215.90]	0.020
**Stage**	Stage I	1	–	–
	Stage II	0.38	[0.14–1.07]	0.068
	Stage III	0.03	[0.02–4.57]	0.389
	Stage IV	1.60	[0.07–32.28]	0.757

## Discussion

Differentiated thyroid cancers of follicular origin generally have excellent outcomes, with 10-year overall survival rates of 80–95% (10). Numerous studies have identified prognostic factors influencing survival and remission, and our findings align with much of this literature.

Female sex was a strong predictor of remission, consistent with prior studies linking male sex to advanced stage and aggressive histology (11,12). Younger age (<55 years) was also associated with higher remission, supporting previous evidence that older patients have higher risks of recurrence and mortality (13–15).

Histological subtype played a significant role: PTC was associated with higher remission rates than FTC, in agreement with earlier reports (1,16). Tumor size ≤4 cm and unilaterality were also favorable factors, consistent with studies that identified larger tumors and bilaterality as predictors of persistence and recurrence (1,17–20).

The absence of extrathyroidal and vascular invasion strongly predicted remission, supporting prior work identifying these features as markers of aggressive disease (21–23). Postoperative TG levels also proved highly predictive, consistent with evidence linking undetectable TG with remission and lower recurrence risk (25,26).

Finally, lymph node and distant metastases significantly reduced the likelihood of remission, in line with studies showing their importance in persistence and recurrence risk (8,11,18). Interestingly, disease stage was not independently predictive in multivariate analysis, likely due to the low frequency of advanced-stage cases.

Despite its retrospective design and some missing data, this study benefits from a large cohort and long follow-up, offering valuable insight into prognostic factors in Moroccan patients with DTC.

DTCs are among the most curable cancers, with high rates of complete remission. This study identified ten predictive factors for remission in Moroccan patients: female sex, younger age, papillary histology, tumor size ≤4 cm, unilaterality, absence of extrathyroidal and vascular invasion, absence of lymph node and distant metastases, and postoperative TG ≤10 ng/mL. Integrating these factors into clinical decision-making can optimize therapeutic strategies, improve outcomes, and help tailor follow-up intensity to patient risk profiles.
